# Knowledge, feelings, and willingness to use palliative care in cancer patients with hematologic malignancies and solid tumors: a prospective, cross-sectional study in a comprehensive cancer center in Germany

**DOI:** 10.1007/s00520-023-07914-0

**Published:** 2023-07-06

**Authors:** Cordula Gebel, Judith Basten, Isabel Kruschel, Thomas Ernst, Ulrich Wedding

**Affiliations:** 1 Department of Palliative Care, Jena University Hospital, Jena, Germany; 2 University Tumor Center, Jena University Hospital, Jena, Germany

**Keywords:** Cancer, Hematologic malignancies, Palliative care, Knowledge, Willingness to use, Feelings

## Abstract

**Purpose:**

Patients with hematologic malignancies (HM) receive palliative care (PC) less often and later than patients with solid tumors (ST). Patients’ lack of knowledge about PC and negative feelings about PC are barriers to their willingness to use PC. Is there a difference between patients with HM and ST in their knowledge and willingness to use PC?

**Methods:**

Two hundred ten patients (85 HM, 125 ST) from an oncology day clinic at a university hospital participated in this cross-sectional, questionnaire-based survey.

**Results:**

Patients with HM and ST had high knowledge and mainly positive feelings about PC. More than half of the patients answered that they would feel reassured by the use of PC, and one-third would feel anxious or hopeless. The majority of patients (58.3%) were willing to use PC. There are no significant differences between patients with HM and ST. In multiple regression analysis, perceived chance of cure and feelings of reassurance and anxiety are associated with willingness to use PC, but not with the HM/ST disease group. More than half (53.9%) of the participants would like the treating physician to choose the timing of a discussion about PC.

**Conclusion:**

Our study shows a high level of knowledge and relatively positive feelings of patients about PC, with no differences between patients with HM or ST. They expect their treating physician to initiate communication about PC. Communication should include the patient’s feelings about PC and their chances of a cure.

**Supplementary Information:**

The online version contains supplementary material available at 10.1007/s00520-023-07914-0.

## Introduction

Several studies have shown that palliative care (PC) can lead to improvements in quality of life and symptom burden in life-limiting illnesses, especially when integrated early in the treatment of cancer [1–3]. Although patients with hematologic malignancies (HM) have a similar symptom burden as patients with solid tumors (ST) in the last months of life [4, 5] and would benefit from PC similarly [6], they receive PC less frequently and later than patients with ST [4, 7–9]. There are several reasons for these differences, such as the heterogeneity of illness trajectories and the different treatment options that can be provided until close to death, and the dependence on transfusions and antibiotics, which also complicate hospice care [10–13].

However, barriers to use PC exist on the part of patients, families, health professionals, and health system structures [14, 15]. The key determinants of willingness to use [16–19] are patients’ knowledge of available options, attitudes, or beliefs about specific treatments. More attention should be paid to these [10]. Previous research has summarized cognitive barriers as poor understanding of PC, misconceptions, and negative attitudes and feelings about PC [9, 13, 20].

Surveys in the general population have shown a lack of knowledge and prejudice about PC across different countries [16, 21–25]. Surveys with cancer patients showed a wide range of low to high knowledge regarding PC [19, 26–30]. Among patients with blood cancers, a recent study by Filippou et al. even showed a high level of understanding of PC [28]. One study reported relatively positive feelings of cancer patients towards PC [27]. Variables associated with a higher level of knowledge and awareness of PC were female gender, higher level of education and socioeconomic status, older age, experience with relatives and friends, working in the health care system, and a close relationship with the treating physician [21, 22, 24, 29, 31].

More studies compared HM specialists and ST specialists than patients with HM or ST regarding attitudes, beliefs, and perceptions about PC [32–34]. Only a few studies have specifically compared knowledge and feelings about PC in patients with ST and HM. Therefore, this study aimed to compare patients with HM and ST in terms of their knowledge and feelings about PC and to investigate whether this can explain parts of the willingness to use PC. Furthermore, it was analyzed which factors influence the willingness to use PC from the patients’ point of view.

## Materials and methods

### Design, setting, and data collection

We conducted a prospective, cross-sectional, single-center, questionnaire-based study of patients attending a hematology and oncology day clinic at a university hospital in Germany. This tertiary care hospital is part of a comprehensive cancer center. The oncology day clinic provides medical and nursing care as well as the possibility of social and psychological consultations. The well-established PC consultation service must be initiated by the treating physician.

Data collection took place from March 2022 to September 2022. Patients were selected using a convenience sampling strategy, where patients were asked by members of the research team to complete the questionnaire once while waiting in the day clinic. To motivate as many patients as possible to participate, the sampling of patients took place several times a week at different times. Adult German-speaking patients with HM or ST who were able to give informed consent were included in this study. Exclusion criteria were insufficient knowledge of German or inability to complete the questionnaire, e.g., due to fatigue or lack of concentration.

This study was performed in line with the principles of the Declaration of Helsinki. Informed consent is available for all participants. The study was approved by the Ethics Committee of the University Hospital Jena, Germany (Reg.-Nr.: 2022–2518-Bef).

### Patients questionnaire

The questionnaire is based on the validated Palliative Care Knowledge Scale (PaCKS) [35]. Further categories included in the questionnaire were developed based on reviews, qualitative and quantitative studies, and clinical expertise [9, 21, 22, 24, 27, 36, 37]. The questionnaire was discussed and agreed upon by the interdisciplinary research team. The final questionnaire consisted of 10 categories. For the present analysis, in addition to demographic and clinical data, 5 categories were analyzed: (a) PC knowledge, (b) feelings regarding PC, (c) points of contact with PC, (d) willingness to use PC, and (e) appropriate timing of PC conversation. A pretest was conducted with 15 people using cognitive interviewing. Some items were adapted based on the pretest. Overall, the questionnaire was found to be acceptable, understandable, and content valid. All items are self-report measures. The questionnaire was in German. For the present publication, all items were translated into English.

Demographic and clinical characteristics: The following demographic data were collected: gender, age group (“18–35”; “36–65”; “66–80”; “ > 80”), educational level (“Hauptschule (secondary school, 5th to 9th grade)”; “Regelschule (secondary school, 5th to 10th grade)”; “Abitur (A-levels),” and migration status (“yes”; “no”). The following clinical information were collected: date of the initial cancer diagnosis, cancer type for ST patients “breast or ovarian or cervical cancer,” “colorectal cancer,” “lung cancer,” “other solid tumors,” and for HM patients “leukemia,” “non-Hodgkin’s lymphoma,” “Hodgkin’s lymphoma,” “multiple myeloma,” and “other hematological malignancies.” In addition, the patients were asked to report their perceived chance of cure as follows: “Cancer diagnoses have different chances of being cured. What is your estimate of your chance of being cured according to the following categories: “00–20%,” “20–40%,” “40–60%,” “60–80%,” and “80–100%.”

#### PC knowledge

Knowledge about PC is collected by the “Palliative Care Knowledge Scale (PaCKS) [35].” The PaCKS includes 13 statements about PC that are rated as true or false. If the answer is correct 1 point is assigned. The total score ranges from 0 to 13, with higher scores representing greater knowledge. A total score of 3 to 6 points is defined as low knowledge, 7 to 10 points as moderate knowledge, and 11 to 13 points as high knowledge of PC [26, 35]. Satisfactory psychometric properties for this questionnaire have been described in the literature [35]. The PaCKS was translated into German according to the “Principles of Good Practice for the Translation and Cultural Adaptation Process for Patient-Reported Outcomes Measures [38].” The translation was authorized by the author, Dr. Kozlov. The item wording can be found in Supplementary Table S1.

#### Feelings regarding PC

According to a study by Chosich et al. feelings of anxiety, loss of hope, and reassurance regarding PC are assessed using 3 items [27]. Anxiety “I would feel anxious if referred to PC.” Loss of hope “I would feel hopeless to be referred to PC.” Reassurance “I would feel reassured through involvement of PC.” These items should be ranked with a 5-point Likert scale from “disagree” to “agree.” These statements are grouped into 3 categories: rejection (disagree /tend to disagree), neutral (part/part), and agreement (agree/tend to agree).

#### Points of contact with PC

Points of contact with PC were assessed with the question: “Which points of contact do you have/had with palliative care?” Multiple choices were allowed for the following categories: “own professional activity in the medical field,” “by relatives,” “by friends,” “voluntary activities,” “conversation with general practitioner,” “conversation with oncologist,” “I actually receive PC,” and “other points of contact.” The categories “by relatives” and “by friends” include cases where either the patient was encouraged in discussions about PC or where the relatives or friends received PC themselves, which allowed the patient to come into contact with PC. It was also possible to indicate if there were “no” points of contact. For group comparison between ST and HM, a variable “at least 1 contact reported” was created.

#### Willingness to use PC

The statement “If I would be diagnosed with a serious illness, I would use palliative care.” was rated on a 5-point Likert scale from “disagree” to “agree.”

#### Timing of PC conversation

This field was addressed with the question: “At what time should a conversation about palliative care take place?” The response categories were as follows: “at no time,” “already before my illness as general information,” “at the time of initial diagnosis,” “at the beginning of treatment,” and “only if the treating physician thinks I need it acutely.”

### Data analysis

The sample size calculation was performed using G*Power version 3.1.9.7 for the comparison of the two patient groups using *t*-test. Defining the significance level at *p* < 0.05 and the power at *w* = 0.95 (95%) resulted in a sample size of 210 patients to detect moderate effects (*d* = 0.50). Statistical analysis was performed with *R* 4.2.1. In addition to the descriptive data analysis (means, standard deviations, frequencies, percentages), inferential statistical procedures (chi-squared tests, Fisher’s exact test, *t*-test) were performed depending on the scale level and the test requirements for the group comparisons ST vs. HM. For the analysis of bivariate relationships, Pearson correlation was used for continuous variables and dot-biserial correlation for dichotomous variables. A multiple linear regression analysis was performed to analyze which factors are significant for the willingness to use PC. In regression analysis, the following predictive factors enter in the field of disease (HM/ST, disease duration, perceived chance of cure), in the field of demographic data (age, gender, educational level), in the field of PC knowledge (total score PaCKS), further in the feelings (anxiety, loss of hope, and reassurance), and own points of contact with PC. Ordinal scaled items were dichotomized for correlation and regression analysis (category: age 0 =  < 65 years; 1 =  > 66 years, educational level 0 = no A-levels; 1 = A-levels). A significance level of *p* < 0.05 was defined for all tests. Missing values were excluded pairwise.

## Results

**Fig. 1:**
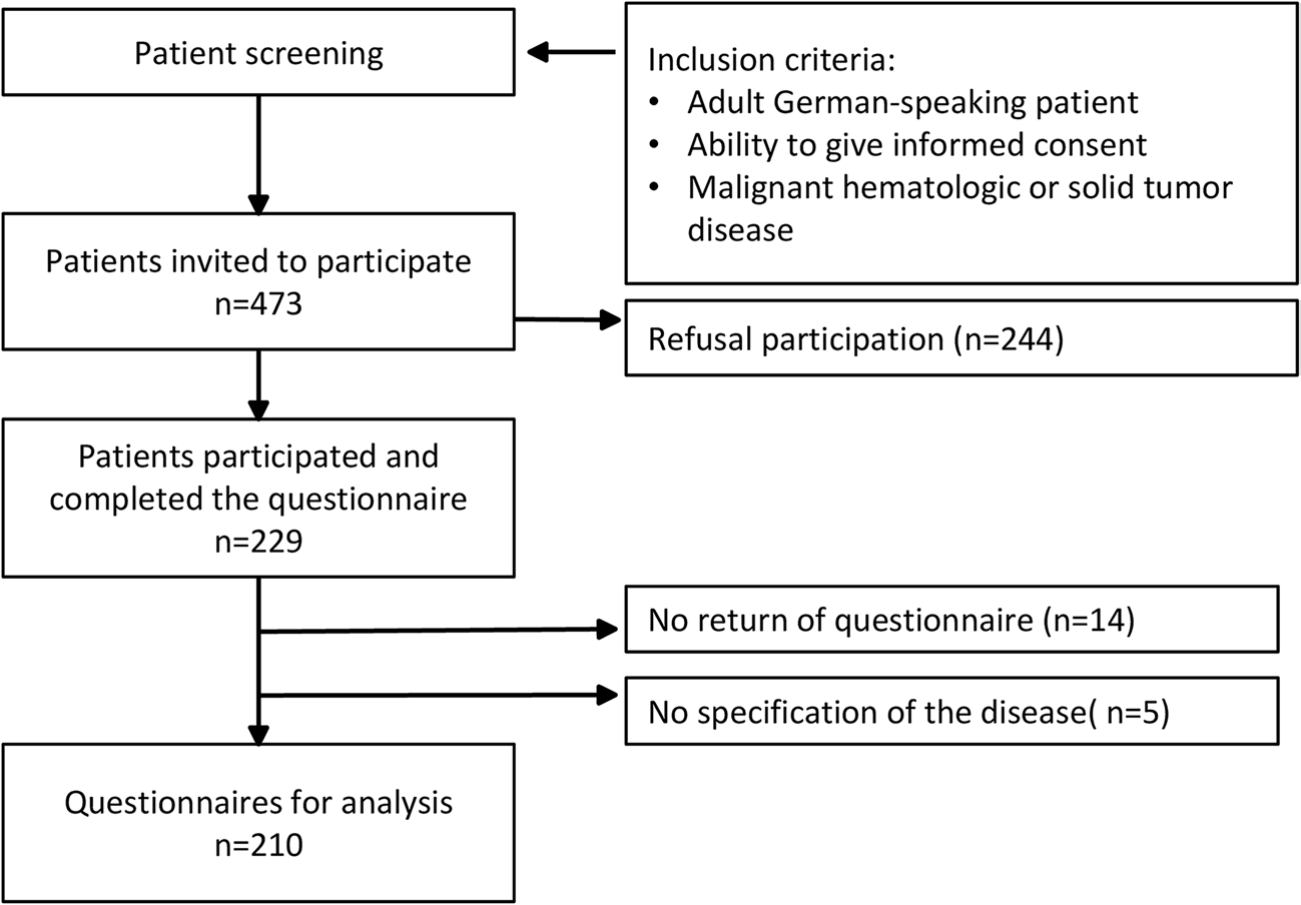
Flowchart data collection

During the study period, 473 patients were invited to participate and 229 agreed, of which 210 were included in the data analysis (Fig. 1). A non-responder analysis cannot be performed due to missing demographic and clinical data of non-participating patients. No systematic data were collected on the reasons for non-participation. Reasons for non-participation, based on feedback from study staff, included the following patients reported experiencing physical, psychological, and cognitive symptoms that would have affected their ability to complete the questionnaire. Other patients were burdened by their medical treatment. In addition, some patients reported a lack of interest in participating in research studies. Demographic and clinical characteristics are summarized in Table 1. Eighty-five patients with HM and 125 patients with ST participated in this study. In total, 52.6% of the participants were female, and 57.4% were younger than 66 years. On average, participants had cancer for M = 3.64 (SD = 4.4) years, with 45.1% having cancer for less than 2 years. Demographic and clinical data did not differ significantly between HM and ST patients.

**Table 1:** Demographic and clinical characteristics

	Total	Patients with hematologic malignancies (HM)	Patients with solid tumors (ST)	Test of significance
Characteristics	*n* (Col, %)	*n* (Col, %)	*n* (Col, %)	*p*-value
Population	210	85 (40.5)	125 (59.5)	
Gender				
Female	110 (52.6)	38 (45.2)	72 (57.6)	0.11^a^
Male	99 (47.4)	46 (54.8)	53 (42.4)	
Missing	1	1	0	
Age group				
18–35	9 (4.3)	4 (4.8)	5 (4.0)	0.14^b^
36–65	111 (53.1)	40 (47.6)	71 (56.8)	
66–80	76 (36.4)	31 (36.9)	45 (36.0)	
> 80	13 (6.2)	9 (10.7)	4 (3.2)	
Missing	1	1	0	
Educational level				
Hauptschule (secondary school, 5th to 9th grade)	26 (12.4)	14 (16.7)	12 (9.6)	0.30^a^
Regelschule (secondary school, 5th to 10th grade)	118 (56.5)	46 (54.8)	72 (57.6)	
Abitur (A-levels)	65 (31.1)	24 (28.6)	41 (32.8)	
Missing	1	1	0	
Migration status				
Yes	4 (2.1)	2 (2.6)	2 (1.7)	1.00^b^
No	189 (97.3)	75 (97.4)	114 (98.3)	
Missing	17	8	9	
Cancer type				
Leukemia		24(28.2)		
Non-hodgkin lymphoma		9(10.6)		
Hodgkin lymphoma		9(10.6)		
Multiple myeloma		31(36.5)		
Other HM		12 (14.1)		
Breast/ovarian/cervical			45 (36.3)	
Colorectal			24(19.2)	
Lung			16(12.8)	
Other ST			40 (32.0)	
Time since initial diagnosis in years (M/SD) (*n* = 195)	3.64 (4.4)	3.60 (4.0)	3.71 (4.7)	0.92^c^
Perceived chance of cure *				
00–20%	57 (31.0)	22 (30.6)	35 (31.3)	0.54^a^
20–40%	16 (8.7)	7 (9.7)	9(8.0)	
40–60%	30(16.3)	11(15.3)	19(17.0)	
60–80%	32(17.4)	9(12.)	23(20.5)	
80–100%	49(26.6)	23(32.0)	26(23.2)	
Missing	26	13	13	

Note. Relative percentage, without missing values, *p*-values based on ^a^chi-squared tests, ^b^Fisher’s exact test, and ^c^*t*-test. *Item: “Cancer diagnoses have different chances of being cured. What is your estimate of your chance of being cured according to the following categories”

### Knowledge and feelings regarding PC

**Fig. 2:**
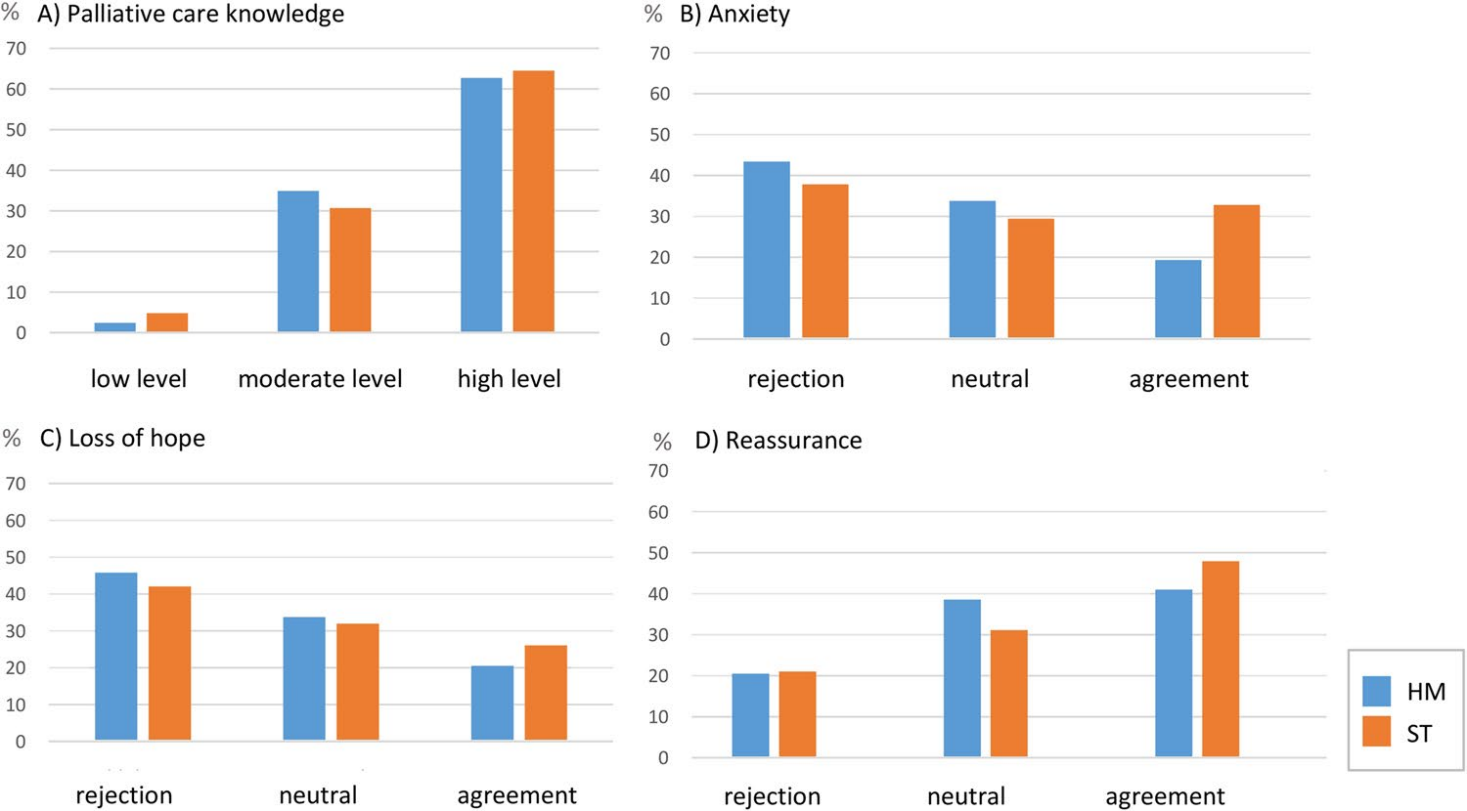
Knowledge and feelings regarding palliative care. **A** Categories of Palliative Care Knowledge Scale (PaCKS); **B** “I would feel anxious if referred to PC.”; **C** “I would feel hopeless to be referred to PC”; **D** “I would feel reassured through involvement of PC”; labels: rejection (disagree /tend to disagree), neutral (part/part), agreement (agree/tend to agree); relative percentage based on disease group, without missing values

There is a high level of PC knowledge in the sample (HM: M (SD) = 10.67 (1.99), ST: M (SD) = 10.88 (1.96), *p* < 0.05), which does not differ significantly between HM and ST. Figure 2A shows a low level of PC knowledge in patient groups HM/ST 2.4%/4.8%, a moderate level in 34.9%/30.7%, and a high level in 62.7%/64.5%. Results for individual items are shown in Supplementary Table S1.

Figures 2 B–C show the distribution of responses regarding feelings about PC. The participants state that they tend to feel more reassured by the inclusion of PC (M = 3.36, SD = 1.30). 45.1% agreed with the statement. In total, 23.8% state “to feel loss of hope” and 43.6% state “not to feel hopeless” (M = 2.64, SD = 1.29). In total, 27.6% would feel anxiety if they were referred to PC. The mean of M = 2.74 (SD = 1.37) shows that the participants are quite neutral about this statement. There are no significant differences between HM and ST in the estimation of feelings towards PC. Means of the scales and test statistics are summarized in Supplementary Table S2

### Willingness to use PC and points of contact

The majority of participants would use PC in the event of a serious illness. In total, 58.3% tend to agree or agree with this statement. In total, 12.6% would not use PC. As shown in Table 2, there was no significant difference between HM and ST in their willingness to use PC. Respondents with HM and ST differ significantly in their points of contact with PC. Patients with ST are significantly more likely to have at least one point of contact with PC than patients with HM (62.6% vs. 24.2%). Differences are about the conversation with oncologists. There are major differences between HM and ST. While 8.3% of patients with HM had a conversation about PC with the oncologist, 19.5% of patients with ST had such a conversation. Regarding current palliative treatment, the difference between HM and ST is similarly large. In total, 2.4% of patients with HM and 13.8% of patients with ST were receiving PC at the time the questionnaire was completed.

**Table 2:** Willingness to use palliative care and points of contact with palliative care

	Total	Patients with hematologic malignancies (HM)	Patients with solid tumors (ST)	Test of significance
	*n* (Col, %)	*n* (Col, %)	*n* (Col, %)	*p*-value
Willingness to use palliative care If I would be diagnosed with a serious illness, I would use palliative care. (M/SD)	3.74 (1.24)	3.71 (1.19)	3.77 (1.28)	0.74^c^
Disagree (1)	18 (8.7)	6 (7.3)	12 (9.7)	
Tend to disagree (2)	8 (3.9)	3 (3.6)	5 (4.0)	
Part/part (3)	60 (29.1)	28 (34.2)	32 (25.8)	
Tend to agree (4)	43 (20.9)	17 (20.7)	26 (21.0)	
Agree (5)	77 (37.4)	28 (34.2)	49 (39.5)	
Missing	4	3	1	
Points of contact with palliative care Which points of contact do you have/had with palliative care? (Multiple choices possible)				
o Own professional activity in medical field	14 (6.8)	7 (8.3)	7 (5.7)	0.64^a^
o By relatives	35 (16.9)	14 (16.7)	21 (17.1)	1.00^a^
o By friends	27 (13.0)	9 (10.7)	18 (14.6)	0.54^a^
o Voluntary activities	3 (1.5)	2(2.4)	1 (0.8)	0.52^b^
o Conversation with a general practitioner	19 (9.2)	5 (6.0)	14 (11.4)	0.28a
o Conversation with a oncologist/hematologist	31 (15.0)	7 ( 8.3)	24 (19.5)	0.04^a^
o I actually receive palliative care	19 (9.2)	2 (2.4)	17 (13.8)	0.00^b^
o other points of contact	3(1.5)	0	3 (2.4)	0.40^b^
At least 1 contact reported	115 (55.6)	38 (45.2)	77 (62.6)	0.02^a^
“No” points of contact	92 (44.4)	46 (57.8)	46 (37.4)	
Missing	3	1	2	

Note. Relative percentage, without missing values, *p*-values are based on ^a^chi-squared tests, ^b^Fisher’s exact test, and ^c^*t*-test

### Factors influencing the willingness to use PC

Table 3 shows the results of the multiple regression analysis. In the multiple regression model, 3 factors are important for predicting the willingness to use PC. Regarding feelings, lower anxiety (*β* =  − 0.26, (− 0.48, − 0.05), *p* < 0.05) and greater reassurance (*β* = 0.23, (0.09, 0.38), *p* < 0.05) are associated with PC involvement. In the field of disease, a lower perceived chance of cure (*β* =  − 0.15, (− 0.30, − 0.00), *p* < 0.05) matters. Patients’ disease type, the period since initial diagnosis as well as demographic data or points of contact do not matter regarding the willingness to use PC.

**Table 3:** Multiple linear regression analysis willingness to use palliative care

Section	Coefficients	*β*	95% CI	*t*-value	p-value
	Intercept			4.23	0.00*
Palliative care knowledge	Palliative Care Knowledge Scale (total score)	0.09	(− 0.06, 0.24)	1.21	0.23
Disease	ST vs. HM^a^	0.04	(− 0.10, 0.18)	0.53	0.60
	Time since initial diagnosis	− 0.04	(− 0.19, 0.10)	− 0.58	0.56
	Perceived chance of cure	− 0.15	(− 0.30, − 0.00)	− 2.03	0.04*
Feelings	Anxiety^b^	− 0.26	(− 0.48, − 0.05)	− 2.42	0.02*
	Loss of hope^b^	0.05	(− 0.16, 0.26)	0.49	0.62
	Reassurance^b^	0.23	(0.09, 0.38)	3.19	0.00*
Points of contact	Points of contact with palliative care^c^	0.13	(− 0.02, 0.28)	1.75	0.08
Demographics	Age category^d^	− 0.05	(− 0.19, 0.09)	− 0.70	0.48
	Gender^e^	0.09	(− 0.05, 0.23)	1.27	0.21
	Educational level category^f^	0.01	(− 0.13, 0.15)	0.15	0.88

Note. There is no multicollinearity (all VIF values less than 3)., *β* standardized regression coefficient, *CI* 95% confidence interval of coefficient *β*; (a) ST = 0, HM = 1; (b) disagree (1) to agree (5); (c) no points of contact = 0, at least 1 contact reported = 1; (d) under 66 years = 1, other = 1; (e) male = 0, female = 1; (f) no A-levels = 0, A-levels = 1; the model fit includes the fit of the whole regression model with all coefficients. *R*^2^, coefficient of determination; adj. = adjusted

Model fit: *N* = 170; *R*^2^ = 0.27; *adj.R*^2^ = 0.22; *F* (11, 158) = 5.21; *p* < 0.05

The Supplementary Table S3 shows that there is a small to moderately significant correlation between the amount of knowledge about PC and the willingness to use PC, the perceived chance of cure with the feelings of anxiety, reassurance, and loss of hope, and also with the point of contact to PC. In addition, in the case of PC knowledge, there is a significant correlation with the categorized education level. Some of the bivariate correlations are no longer present after adjustment for sociodemographic variables and other predictors, as shown in the multiple regression analysis.

### Timing of PC conversation

**Fig. 3:**
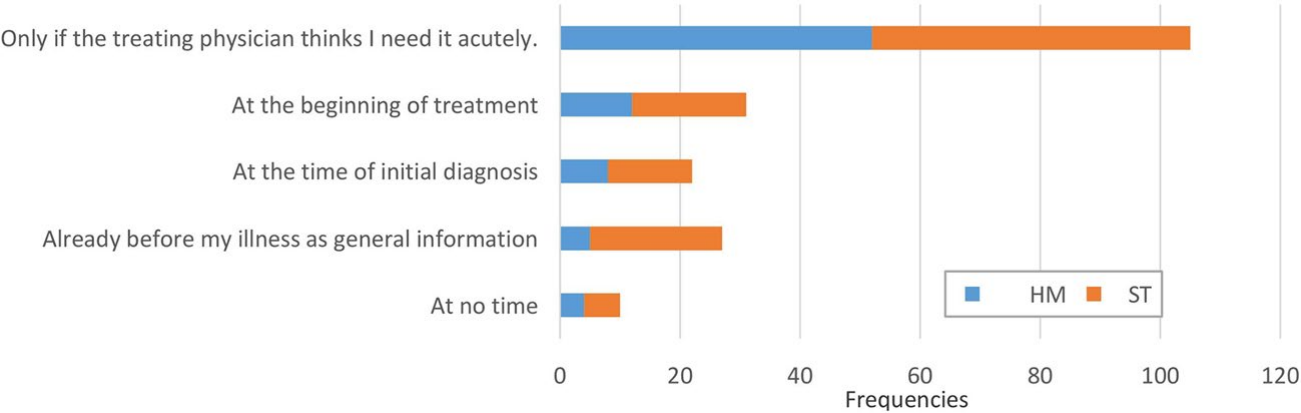
At what time should a conversation about palliative care take place?

As shown in Fig. 3, half of the participants (53.9%) would like the conversation regarding PC to be initiated by the treating physician. Only 5.1% of the participants state that they do not want to have such a conversation. In total, 13.8% of participants would like to have a conversation about PC at the beginning of treatment, 11.3% at the time of initial diagnosis, and 15.9% would like to have a conversation about this topic before a serious illness for general education. The HM and ST groups were not significantly different (*X*^2^ (4) = 9.0, *p* > 0.05).

## Discussion

Our survey showed a high level of knowledge regarding PC among patients with HM and ST, with no significant difference between these two groups. Both groups have quite positive feelings regarding PC, and the majority of patients are willing to use PC. Important factors influencing the willingness to use PC are a low perceived chance of cure and higher feelings of reassurance and lower levels of anxiety. More than half of the participants would like a conversation about PC to be initiated by the attending physician.

According to Ogunsanya et al. (2021), cancer patients are more likely to have more knowledge about PC compared to the general population [24]. In contrast, a recent study conducted in Iran showed low to moderate knowledge of PC [26]. Considering cancer patients over 65 years of age in the USA, this group also showed low knowledge and a lack of understanding [19]. For the interpretation of these results, it is necessary to focus on social contextual factors and the level of implementation of PC in the health care system. A representative population survey in Germany shows an increase in knowledge of the term “palliative care” from 49% of participants in 2012 to 79% of participants in 2022. Also, almost twice as many participants (2012: 31%, 2022: 58%) were able to correctly define “palliative [39].” It can therefore be assumed that the level of knowledge in Germany is already increasing in the general population.

In our study, in contrast to other studies [19, 27, 28], there was no effect of age or gender on PC knowledge or willingness to use PC. Only educational level showed a significant correlation with PC knowledge. This may be related to the composition of the sample. Compared to the general German population, our study population was better educated, included more women, and only four persons reported a migration status [40]. The results should be interpreted with this in mind. It is possible that patients with a lower educational level and patients with a migrant status need more and different information regarding PC.

Like in other studies, positive feelings about PC are associated with knowledge and also with willingness to use PC. These correlations have been demonstrated in patients undergoing hematopoietic stem cell transplantation [29, 30], as well as in the general population [41], and should be the focus of outreach and education activities.

Despite the lack of differences observed among patients with HM and ST in terms of knowledge, feelings regarding PC, and perceived chance of cure, there are significant disparities in their contact with treating physicians. HM patients had fewer conversations with their treating physicians and received fewer referrals to PC. More than half of the participants in our study would potentially use PC, similar to the study by Filippou et al. (2022) [28]. There is no difference between patients with HM and ST. As reported in the literature [4, 7–9], it appears that significantly fewer patients with HM than with ST receive PC. Utilization rates are lower than in other studies of cancer patients; for example, Chosich et al. in Australia reported 21% of patients receiving PC. In the study by Filippou et al. (2022), 6.5% of patients with HM received a PC consultation. The cohort analyzed had a high level of education and PC knowledge, more positive feelings about PC, and similar perceived chances of cure, and even in this sample there are large differences in PC co-treatment. As in Filippou’s study, less than 10% of patients with HM reported having discussed PC with their hematologist. This is in contrast to patients with ST, where conversations with oncologists about PC are more than twice as common [28]. Given that the vast majority of patients report that the treating physician should decide when a PC discussion takes place, there is a need for intervention. Two strategic directions are possible. On the one hand, it might be useful to educate oncologists about patients’ expectations and how to address relevant PC topics communicatively. On the other hand, patients could be emphasized to talk about these topics themselves to express patient autonomy or at least to inform the treating physician to initiate a conversation about PC if needed. The presented data may relieve oncologists of the fear of taking away hope by mentioning PC, as many patients do not associate PC with loss of hope [30, 42–44].

There is a positive correlation between PC knowledge and willingness to use PC. However, this correlation is no longer relevant when other factors are included in the multiple regression analysis. However, this does not mean that PC knowledge is irrelevant. As there is already a high level of PC knowledge in the present sample, PC knowledge explains little additional variance. As well as shown in the regression analysis for willingness to use, only feelings regarding PC and the perceived chance of cure are relevant. This underlines the need to include feelings in the discussion with the patient, in addition to providing medical information, to increase the willingness to use PC and to enable the patient to make an appropriate decision.

## Strengths and limitations

To our knowledge, this is the first survey comparing knowledge, feelings, and willingness to use PC between HM and ST in Germany. It shows that there are no major differences in the estimated outcomes between patients with HM and ST. The interpretation of the results is restricted by several limitations. Participants who were unable to complete the questionnaire, such as individuals experiencing fatigue, were excluded from the data collection process. The sampling bias is also related to gender, education, migration status, and age and therefore cannot be generalized to all cancer patients. In addition, this survey was conducted in a comprehensive cancer center with an established PC team and may not be generalizable to other centers. As only one center is involved, the comparison of patients with HM and ST is meaningful because they are subject to the same contextual conditions. Another limitation of our study is the use of a questionnaire validated in English. This may limit the generalizability of the results, even though we translated the questionnaire for our study into German professionally and carefully, following a standard procedure. The calculated sample size of 210 subjects is large enough to detect moderate effects/differences between the two patient groups of HM and ST. A non-significant result, therefore, only means that there are no mean differences between the groups. We chose this criterion because if there were small differences between the groups, they would be less relevant in clinical practice. The entire study is based on self-reports. It is therefore not possible to validate clinical data, such as the chance of cure, with other data. It is important to note that in other studies, cancer patients tended to overestimate their chances of being cured; there is no information about this in the present study [44, 45].

## Conclusion

In our patient cohort, patients with HM and ST show a relatively high level of knowledge and openness to PC. The treating physicians play an important role in the incorporation of PC into treatment. The vast majority of patients would like the treating physician to decide when to discuss PC. To increase willingness to use PC, it is necessary to address feelings in addition to knowledge. Further research should address the knowledge and feelings of family members, as they contribute significantly to decision-making.

## Supplementary Information

Below is the link to the electronic supplementary material.

Supplementary file1 (DOCX 30.8 KB)

## Data Availability

The data that support the findings of this study are available from the corresponding author, upon reasonable request.
