# Supplementary material for: Knowledge, feelings, and willingness to use palliative care in cancer patients with hematologic malignancies and solid tumors: a prospective, cross-sectional study in a comprehensive cancer center in Germany
Source: Support Care Cancer. 2023 Jul 6;31(7):445. doi: 10.1007/s00520-023-07914-0 (PMC10326137; doi:10.1007/s00520-023-07914-0)
Supplement: Supplementary file 1 — Supplementary file1 (DOCX 30.8 KB) [file 520_2023_7914_MOESM1_ESM.docx]

**Online-Supplement**

Gebel C, Basten JE, Kruschel I, Ernst T; Wedding U (2023). Knowledge, feelings and willingness to use palliative care in cancer patients with hematologic malignancies and solid tumors. A prospective, cross-sectional study in a comprehensive cancer center in Germany.

**Corresponding author:** Cordula Gebel: cordula.gebel@med.uni-jena.de

ORCID: 0000-0002-8305-9176

**Online-Supplement**

**Table S1** Patient responses to the Palliative Care Knowledge Scale (PaCKS) Items

**Table S2** Knowledge and feelings regarding palliative care

**Table S3** Correlation matrix of willingness to use palliative care, palliative care knowledge and influencing factors.

**Table S1** Patient responses to the Palliative Care Knowledge Scale (PaCKS) Items

|  |  |  | Total | Patients with hematologic malignancies (HM) | Patients with solid tumors  (ST) |
| --- | --- | --- | --- | --- | --- |
| Item | Correct response | n | M (SD) | M(SD) | M(SD) |
| One goal of palliative care is to address any psychological issues brought up by serious illness. | true | 205 | 0.79 (0.41) | 0.78 (0.41) | 0.80 (0.41) |
| Stress from serious illness can be addressed by palliative care. | true | 205 | 0.75 (0.43) | 0.77 (0.42) | 0.74 (0.44) |
| Palliative care can help people manage the side effects of their medical treatments. | true | 202 | 0.84 (0.37) | 0.78 (0.41) | 0.87 (0.33) |
| When people receive palliative care, they must give up their other doctors. | false | 204 | 0.91 (0.29) | 0.94 (0.24) | 0.89 (0.32) |
| Palliative care is exclusively for people who are in the last six months of life. | false | 206 | 0.88 (0.32) | 0.87 (0.34) | 0.89 (0.31) |
| Palliative care is specifically for people with cancer. | false | 205 | 0.8 (0.16) | 0.79 (0.41) | 0.8 (0.4) |
| People must be in the hospital to receive palliative care. | false | 205 | 0.83 (0.37) | 0.8 (0.41) | 0.86 (0.35) |
| Palliative care is designed specifically for older adults. | false | 206 | 0.93 (0.25) | 0.91 (0.28) | 0.94 (0.23) |
| Palliative care is a team-based approach to care. | true | 206 | 0.93 (0.25) | 0.96 (0.19) | 0.91 (0.29) |
| One goal of palliative care is to help people better understand their treatment options. | true | 206 | 0.76 (0.43) | 0.76 (0.43) | 0.76 (0.43) |
| Palliative care encourages people to stop treatments aimed at curing their illness. | false | 206 | 0.86 (0.35) | 0.87 (0.34) | 0.85 (0.35) |
| One goal of palliative care is to improve a person’s ability to participate in daily activities. | true | 205 | 0.69 (0.46) | 0.62 (0.49) | 0.73 (0.44) |
| Palliative care helps the whole family cope with a serious illness. | true | 206 | 0.92 (0.27) | 0.89 (0.31) | 0.94(0.23) |

**Table S2** Knowledge and feelings regarding palliative care

|  |  |  | Total | Patients with hematologic malignancies (HM) | Patients with solid tumors  (ST) | Test of Significance | |
| --- | --- | --- | --- | --- | --- | --- | --- |
| Scale/Item | Specification | n | M (SD) | M (SD) | M (SD) | T(df) | p-Wert |
| **Palliative Care Knowledge Scale** (PaCKS - Total Score)* | Number of correct answers | 207 | 10.80 (1.97) | 10.67  (1.99) | 10.88 (1.96) | -0.73 (205) | 0.47 |
| **Anxiety** “I would feel anxious if referred to palliative care." | Disagree (1) to agree (5) | 199 | 2.74  (1.37) | 2.51  (1.29) | 2.90  ( 1.40) | -1.97 (197) | 0.05 |
| **Loss of hope** “I would feel hopeless to be referred to palliative care”. | Disagree (1) to agree (5) | 202 | 2.64  (1.29) | 2.52  (1.19) | 2.73  (1.34) | -1.16 (200) | 0.25 |
| **Reassurance** “I would feel reassured through involvement of palliative care”. | Disagree (1) to agree (5) | 202 | 3.36  (1.30) | 3.29  (1.20) | 3.40  (1.36) | -0.61  (200) | 0.54 |

Note.* All items of the scale, means and standard deviations are listed in Table S1.

**Table S3** Correlation matrix of willingness to use palliative care, palliative care knowledge and influencing factors.

| Variable | 1 | 2 | 3 | 4 | 5 | 6 | 7 | 8 | 9 | 10 | 11 |
| --- | --- | --- | --- | --- | --- | --- | --- | --- | --- | --- | --- |
|  |  |  |  |  |  |  |  |  |  |  |  |
| 1. Willingness to use palliative care | 1.00 |  |  |  |  |  |  |  |  |  |  |
|  |  |  |  |  |  |  |  |  |  |  |  |
| 2. Palliative Care Knowledge Scale (Total Score) | .24* |  |  |  |  |  |  |  |  |  |  |
| 3. ST vs. HM (a) | -.02 | -.05 |  |  |  |  |  |  |  |  |  |
| 4. Time since initial diagnosis | .07 | .02 | -.01 |  |  |  |  |  |  |  |  |
| 5. Percieved chance of cure | -.25* | -.15* | .03 | -.22* |  |  |  |  |  |  |  |
| 6. Anxiety (b | -.32* | -.28* | -.14 | -.01 | .25* |  |  |  |  |  |  |
| 7. Loss of hope (b) | -.23* | -.28* | -.08 | .01 | .11 | .75* |  |  |  |  |  |
| 8. Reassurance (b) | .33* | .18* | -.04 | .17* | -.15* | -.25* | -.25* |  |  |  |  |
| 9. Points of contact with palliative care (c) | .22* | .31* | -.17* | .24* | -.13 | -.17* | -.18* | .19* |  |  |  |
| 10. Age category (d) | -.07 | -.13 | .08 | .13 | -.05 | -.08 | -.05 | .05 | -.09 |  |  |
| 11. Gender (e) | .09 | .03 | -.12 | .13 | -.12 | .19* | .11 | .03 | .03 | .04 |  |
| 12. Educational level category (f) | .02 | .19* | -.04 | .06 | .08 | -.07 | -.17* | .01 | .02 | -.04 | -.00 |

*Note* Pearson correlation was determined for continuous variables and dot-biserial correlation for dichotomous variables. * indicates *p* < 0.05; a) ST=0, HM=1; (b) disagree (1) to agree (5); c) no points of contact=0, at least 1 contact reported =1; d) under 66 years=1, other=1; e) male=0, female=1; f) no A-levels= 0, A-levels=1
